# Does the Degree of Prematurity Relate to the *Bayley-4* Scores Earned by Matched Samples of Infants and Toddlers across the Cognitive, Language, and Motor Domains?

**DOI:** 10.3390/jintelligence11110213

**Published:** 2023-11-08

**Authors:** Emily L. Winter, Jacqueline M. Caemmerer, Sierra M. Trudel, Johanna deLeyer-Tiarks, Melissa A. Bray, Brittany A. Dale, Alan S. Kaufman

**Affiliations:** 1 School of Health Sciences Clinical PsyD Program, Touro University, New York, NY 10036, USA; 2 Department of Educational Psychology, University of Connecticut, Storrs, CT 06268, USA; jacqueline.caemmerer@uconn.edu (J.M.C.); melissa.bray@uconn.edu (M.A.B.); alan.kaufman@uconn.edu (A.S.K.); 3 Department of Occupational and Environmental Medicine, University of Connecticut School of Medicine, Farmington, CT 06030, USA; sierra.trudel@uconn.edu; 4 School-Clinical Child Psychology Program, Pace University, New York, NY 10038, USA; jdeleyertiarks@pace.edu; 5 Department of Special Education, Ball State University, Muncie, IN 47306, USA; badale@bsu.edu

**Keywords:** *Bayley-4*, early childhood assessment, preterm birth, age differences

## Abstract

The literature on children born prematurely has consistently shown that full-term babies outperform preterm babies by about 12 IQ points, even when tested as adolescents, and this advantage for full-term infants extends to the language and motor domains as well. The results of comprehensive meta-analyses suggest that the degree of prematurity greatly influences later test performance, but these inferences are based on data from an array of separate studies with no control of potential confounding variables such as age. This study analyzed *Bayley-4* data for 66 extremely premature infants and toddlers (<32 weeks), 70 moderately premature children (32–36 weeks), and 133 full-term children. All groups were carefully matched on key background variables by the test publisher during the standardization of the *Bayley-4*. This investigation analyzed data on the five subtests: cognitive, expressive communication, receptive communication, fine motor, and gross motor. A multivariate analysis of covariance (MANCOVA) assessed for group mean differences across the three subsamples, while controlling for the children’s age. Extremely premature children scored significantly lower than moderately premature children on all subtests, and both preterm groups were significantly outscored by the full-term sample across all domains. In each set of comparisons, the cognitive and motor subtests yielded the largest differences, whereas language development, both expressive and receptive, appeared the least impacted by prematurity. A follow-up MANOVA was conducted to examine full-term versus preterm discrepancies on the five subtests for infants (2–17 months) vs. toddlers (18–42 months). For that analysis, the two preterm groups were combined into a single preterm sample, and a significant interaction between the age level and group (full-term vs. preterm) was found. Premature infants scored lower than premature toddlers on receptive communication, fine motor, and cognitive. Neither expressive communication nor gross motor produced significant discrepancies between age groups The findings of this study enrich the preterm literature on the degree of prematurity; the age-based interactions have implications for which abilities are most likely to improve as infants grow into toddlerhood.

## 1. Does the Degree of Prematurity Relate to the *Bayley-4* Scores Earned by Matched Samples of Infants and Toddlers across the Cognitive, Language, and Motor Domains?

Prematurity, when an infant is born before full-term pregnancy (37–40 weeks), impacts one out of every ten births in the United States (Centers for Disease Control and Prevention 2021), while impacting about 15 million babies worldwide (Walani 2020). Nevertheless, differences amongst groups in the United States still exist regarding this statistic, such as the disproportionate impact of preterm birth on Black communities (14.4%), with prematurity rates being 50% higher than babies born to White (9.1%) and Hispanic (9.8%) parents (Centers for Disease Control and Prevention 2021). Further, although there has been an improvement in the United States in terms of preterm birth reduction (Centers for Disease Control and Prevention 2021), worldwide, preterm birth rates are around 11% (Walani 2020). Despite the decrease in U.S. preterm births and improvements made to prenatal (prior to birth) and neonatal (post-birth) care, preterm birth impacts the health and developmental outcomes of babies and families across their lifetimes.

A preterm birth impacts diverse facets of development: physical health, child development, cognition, motor skills, language abilities, and so on (e.g., Brydges et al. 2018; de Kieviet et al. 2009; Woodward et al. 2009). The intensity of the effect of a preterm birth also varies, depending on various factors such as access to timely and effective intervention, how many weeks early the baby was delivered, and access to quality neonatal and prenatal care. Sadly, grave discrepancies exist in the outcomes for preterm births based on the access to quality care and privilege, connecting intimately to the fact that preterm birth accounts for 18% of deaths for children under the age of five as well as 35% of deaths of newborns under the age of 28 days (Walani 2020). At the same time, due to vast improvements in perinatal care, over 95% of babies born prematurely do survive, living into adulthood to live healthy and relatively undisturbed (because of their premature birth status) lives (Crump et al. 2013, 2019; D’Onofrio et al. 2013; Juárez et al. 2016; Raju et al. 2017). However, there are developmental concerns around babies born preterm given their early delivery.

In the sections below, critical areas of development related to the assessment of infant and toddler skills and later outcomes are considered by consulting the *Bayley Scales of Infant and Toddler Development, Fourth Edition* test manual (*Bayley-4*; Bayley and Aylward 2019), along with other sources. In addition, the following domains are assessed: cognition, motor skills (fine and gross), and language (receptive and expressive), with the hope of further contributing to the literature with the updated *Bayley-4*, exploring questions around prematurity severity and age differences using the most current and sophisticated measurement tool available to clinicians. 

## 2. Cognition

### 2.1. Preterm versus Full-Term

The cognitive development in infants and toddlers relies on measuring sensorimotor abilities, exploration, manipulation, object-relatedness, and memory and developing executive functions (Bayley and Aylward 2019). A preterm birth is known to be associated with lower intelligence. This disparity in intelligence is observed in three meta-analyses on the topic (Allotey et al. 2017; Bhutta et al. 2002; Kerr-Wilson et al. 2012). Each meta-analysis included samples tested both in the U.S. and internationally, most notably in the U.K., Canada, Australia, Spain, and Norway. All three meta-analyses implement a variety of intelligence tests, most commonly the Wechsler, Kaufman, and McCarthy tests, and showed consistent evidence, based on decades of quality research investigations: children born prematurely earned IQs on a wide variety of intelligence tests that were, on average, 11–12 points lower than those born full-term (Allotey et al. 2017; Bhutta et al. 2002; Kerr-Wilson et al. 2012). 

The overall findings of lower cognitive scores for premature infants, relative to full-term infants, apply equally to the U.S. and international samples. For example, in Australia, premature children scored 11.9 IQ points lower than full-term children (Cheong et al. 2017), a finding observed as well in New Zealand for children tested at ages 4, 6, 9, and 12 years, who averaged 10 points lower across age groups (Mangin et al. 2017). These deficits have been noted in premature children in Brazil who scored, on average, 4 points lower on standardized IQ measures than their full-term peers did (Maggi et al. 2014); in Sweden, where the deficit averaged 14.2 IQ points (Kaul et al. 2021; Serenius et al. 2016); in Bavaria, where the deficit averaged 7 points among ages 4, 6, 8, and 26 years (Breeman et al. 2015); and in the Netherlands, where a 10-point IQ difference was found between prematurely born/very low birth weight children and term-born (Potharst et al. 2013). In the studies with Dutch children, those born at full term also outscored those born prematurely on processing speed, working memory, and attention (Bogičević et al. 2019; Potharst et al. 2013). 

Luttikhuizen dos Santos et al. (2013) conducted a meta-analysis of 14 Bayley studies (*n* = 1330) that included very premature samples (<33 weeks and/or low birth weight < 1500 g). In each study, infants were tested on the *Bayley-I* or *Bayley-II* at ages 4 to 36 months (most samples were between 12 and 24 months) and later tested again (ages 3 to 14, with a median age of 5 using a comprehensive IQ test, namely, a Wechsler, Binet, Kaufman, or McCarthy). The authors’ primary question was how predictive the Bayley global cognitive and language score (i.e., the Mental Development Index or MDI) was of later intellectual functioning. The results were impressive. The 14 studies showed correlations of the Bayley test with childhood IQ that ranged from 0.39 to 0.73 (mean = 0.61); all but two of the coefficients were >0.50. These results suggest that cognitive abilities measured in infancy and toddlerhood (the age ranges of interest in our study) are remarkably predictive of later IQ, accounting for 37% of the variance. Further, the obtained coefficients for the premature samples were not significantly mediated by birthweight, gestational age, or the interval between assessments. These findings are consistent with recent research (Jaekel et al. 2019). Only a handful of studies demonstrate a lack of preterm cognitive impact, instead highlighting language deficits, which may be due to differing definitions of language or cognitive ability from study to study (Guarini et al. 2009). 

Though comprehensive, the meta-analyses included very few studies on premature children who were tested as infants and toddlers. Because these age groups are the focus of the present study, we found seven studies that compared the cognitive ability of full-term vs. preterm children on the *Bayley-I* (Bode et al. 2009; Censullo 1994; Munck et al. 2010; Sajaniemi et al. 1998); the *Bayley-II* (Gray et al. 2006); and the *Bayley-III* (Anderson 2010; Toome et al. 2012). The total *n* = 2088 (1041 preterm, 1047 full-term) for the seven studies of two-year-olds. Full-term toddlers outscored preterm toddlers on measures of cognitive ability by 8-21 standard-score points (median = 12 points). Some other well-designed Bayley studies on prematurity (e.g., Sanchez et al. 2019; Spencer-Smith et al. 2015) did not relate directly to our research questions. 

In fact, the meta-analyses did not have many samples of preschool children. We developed Table 1 to summarize the findings of studies on children born prematurely. Based on the 11 samples of young children (ages 1.5–4 years) included in Table 1, the full-term children consistently outscored the preterm children across differing measures and at differing ages of testing, by 5.25 to 19.65 IQ points (Median = 12.75). Once again, the effects of prematurity on the cognitive ability of very young children is comparable in magnitude to the effects for school-age children and adolescents.

### 2.2. Cognitive Loss and Degree of Prematurity

Kerr-Wilson et al. (2012) and Allotey et al. (2017) provided interesting data on how the degree of prematurity affects the later intellectual functioning. In the 2012 meta-analyses, the pooled mean average IQ differences in favor of full-term births averaged 13.9 points for infants born extremely prematurely (<28 weeks); 11.4 points for moderate prematurity (29–31 weeks); and 6.4 points for mild prematurity (≥32 weeks). Overall, the findings suggest a linear dose–response relationship, with each additional week of gestation presenting an increase in intelligence. Allotey et al. (2017) also grouped their studies by the degree of prematurity. They found a clear-cut relationship between IQ difference and the number of weeks born early: <28 weeks = 11.7 points, 17 studies; 28–34 weeks = 11.0 points (34 studies); and 34–37 weeks = 3.6 points (5 studies). 

Woodward et al. (2009) studied the degree of prematurity in a group of 4-year-olds who were tested on a four-subtest Wechsler short form as part of a prospective longitudinal investigation in New England. They compared short-form IQs of extremely preterm (23–27 weeks, *n* = 43), very preterm (28–33 weeks, *n* = 62), and full-term (38–41 weeks, *n* = 107) children. Full-term children scored 9.8 points higher than the combined preterm sample, but the two preterm samples displayed only a 1.8-point difference, with the very preterm scoring higher. However, the use of a Wechsler short form, rather than the comprehensive battery, is a methodological limitation of this study. 

In addition, Toome et al. (2012) administered the *Bayley-III* to 155 preterm 2-year-olds in Estonia and to a matched full-term control group. They subdivided the premature sample by the degree of prematurity (<25 weeks, *n* = 17; and 26–31 weeks, *n* = 138). The very small sample size of the extraordinarily premature sample is a methodological limitation of this study; nonetheless, the results are instructive. The full-term sample scored 8.6 standard-score points higher than the total premature sample of 2-year-olds on the *Bayley-III* cognitive test. Within the premature group, the “28–31” group outscored the “<25” group by 7 points, which is consistent with the results of the meta-analyses and particularly relevant to the present study because of the use of the Bayley Scales.

Taken together, researchers who conducted meta-analyses on cognition and prematurity, as well as the few studies that used separate samples of extremely and moderately preterm children, agree: the degree of prematurity is a critical yet underutilized factor to consider in research assessing preterm cognitive abilities, aligning with more recent calls for research to consider differences in cognition based on varying degrees of prematurity (Brydges et al. 2018).

## 3. Motor Skills

### 3.1. Gross Motor Skills

Gross motor skills facilitate large, broad bodily movements (e.g., moving a leg, waving an arm), integrating the coordination of the muscular, skeletal, and nervous systems (Vorvick et al. 2021). Gross motor skills develop in infants prior to fine motor skills. Preterm babies experience delays in gross motor skills until early grade school (Allotey et al. 2017; Ribeiro et al. 2017), and they often have worse gross motor outcomes (*d* = −0.53, 95% CI = −0.60–−0.46, *p* < .001; de Kieviet et al. 2009). These concerns are drastically visible between full- and preterm infants in the first 18 months of life, with preterm male infants having gross motor skill deficits on standardized measures of motor functioning even after correcting for the degree of prematurity (Bayley and Aylward 2019; van Haastert et al. 2006). Practically, infants may often be referred for physical/occupational therapy services within the first year and a half of life due to widespread motor delays. Such motor development abnormalities eventually dissipate as the central nervous system matures, and delays are no longer concerning clinically or practically (Bartlett 2000; Bartlett and Fanning 2003). Research also suggests that motor skills within the first year of life do not accurately show long-term outcomes, with a high percentage of children who initially received abnormal scores achieving average scores by the age of four (Prins et al. 2010). However, Goyen et al. (2011) did find that children’s scores on early childhood assessments of motor skill, including the Peabody Gross and Fine Motor Skills and Griffith’s Locomotor Scales, at ages 3 and 5, accurately predicted the occurrence of a developmental coordination disorder at age 8. Further, current meta-analytic reviews (Allotey et al. 2017) suggest that future research should further explore these differences between fine and gross motor skill development, as research has focused on general motor skill development as opposed to specific motor skills. These differences in “broad motor skills” between individuals born at full term and prematurely have been noted across international samples in China (Lee et al. 2004), Italy (De Rose et al. 2013), and the Netherlands (Van Hus et al. 2014), as well as in Brazil, with an overrepresentation of children born prematurely diagnosed with motor coordination disorders (Maggi et al. 2014). 

### 3.2. Fine Motor Skills

Fine motor skills, which coordinate smaller muscle movements, encompass tasks such as one’s ability to reach, grasp, and manipulate objects using the hand, fingers, and especially the thumb (Cuffaro 2011). Many young infants possess fine motor skill weakness and delays, with many medical professionals and NICU follow-ups adjusting for prematurity until the child is two years of age (Lenke 2003). Later in development, a significant portion of preterm individuals experience continued fine motor skill deficits (Belanger 2021) to around age five (Lee et al. 2016) or even into adolescence (Thomas et al. 2017). In childhood, 40–60% of preterm babies have mild to moderate fine motor concerns (Burns et al. 2004; Salt and Redshaw 2006) or have developmental coordination disorders (Wocadlo and Rieger 2008). In sum, research suggests that preterm infants have fine motor skill deficits compared with their full-term peers (*d* = −0.86, 95% CI = −0.99–−0.73, *p* < .001; de Kieviet et al. 2009; Ribeiro et al. 2017). These findings hold up cross-culturally as well, highlighting results from studies on children from the Netherlands (Potharst et al. 2013) and Australia (Lorefice et al. 2015) for visual–motor integration as well as perceptual–motor abilities (De Rose et al. 2013).

Several studies have confirmed that early motor delays experienced by this population do persist into school age. Among children born preterm, at preschool and early elementary ages, deficits in fine motor, gross motor, and sensorimotor skills are apparent (Goyen et al. 2011; Rademaker et al. 2007). Between the ages of 7–10, children born preterm showed a poorer performance than their full-term counterparts on the *Bruininks–Oseretsky Test of Motor Proficiency* (Svien 2003) and the *Movement Assessment Battery for Children* (Goyen and Lui 2009).

## 4. Language

Cognitive ability correlates with language development and predicts performance on many outcomes, such as the ability to read, decode, and comprehend (Bayley 2006). In a seminal study, preterm infants (less than 34 weeks gestation) who were two years old at testing were screened for language impairments and delays using the *Bayley-III* Scale. Lower cognitive and language composite scores were predictive of each other, especially when using expressive language scores (Mossabeb et al. 2012). 

### 4.1. Expressive Language

Expressive language includes communication through different mediums, such as spoken, written, and body language (Frazier 2011a). The connection between expressive language development and motor control areas of the brain suggests that individuals with average motor development often have average expressive language skills compared with individuals with mild and moderate motor developmental delays (Ross et al. 2018). The overlap in developmental coordination disorders and language deficits in expressive, receptive, mixed, and phonological disorders is active and present (Hill 2001). Differences in expressive communication can be seen at early ages. Extremely premature children (born before 27 weeks gestation) who were assessed at age 2.5 years on the *Bayley-III* had significantly lower scores on expressive language compared with full-term controls (*p* = <0.001). When the sample was broken down to the degree of language delay, however, 54% of the sample did not display expressive communication delays (Månsson and Stjernqvist 2014). Further, school-aged infants born preterm consistently score worse for total language abilities (*d* = 9.64, *p* < .001), receptive (*d* = 5.05, *p* < .001), and expressive (*d* = 5.19, *p* < .001), as well as on phonological awareness (*d* = 6.38, *p* < .001) and syntax (*d* = 2.12, *p* < .03; Zimmerman 2018), highlighting prematurity’s impact on language development. Additionally, research has also indicated that receptive language delays were increasingly common over expressive language delays for children born extremely prematurely (Woodward et al. 2009). The literature on prematurity and language deficits extends to a variety of diverse languages and cultures, such as in Brazil (Verreschi et al. 2020), Chile, Estonia, and Bavaria (Putnick et al. 2017; Tulviste et al. 2020; Varela-Moraga et al. 2023).

### 4.2. Receptive Language 

Receptive language is a classification of language consisting of a person’s ability to understand what is communicated via spoken, written, and nonverbal, including signed language (Frazier 2011b). Receptive language is the ability to comprehend others’ communication. Prior research echoes findings that the overall language may be impaired for preterm individuals, suggesting an impairment in receptive-specific skills (Zimmerman 2018). Findings from Månsson and Stjernqvist (2014) also indicated receptive language delays similar to expressive language, as did findings from Da Costa Ribeiro et al. (2016) in Brazil. However, developmental delays were less extreme in language than in cognitive and motor subtests. For example, 60% of the sample did not show a delay in receptive language skills despite their level of prematurity. Conflicting findings emerge, with some studies suggesting that prematurity is not connected to receptive vocabulary deficits, suggesting that the degree to which an individual is born prematurely does not significantly predict their ability to understand words (*M* = 108.6, *SD* = 15.4, *p* > .05; Lee et al. 2011). 

### 4.3. Overall Language Ability

Woodward et al. (2009) administered the *Clinical Evaluation of Language Fundamentals Test* (*CELF*; Wiig et al. 2013) to the same 4-year-olds who were tested on a four-subtest Wechsler short form. The 105 preterm children (43 extremely, 62 very) scored substantially lower than the 107 full-term children on the *CELF* measures of both receptive language (6.8 standard-score points) and expressive language (5.5 points). However, as was true for the cognitive ability in this sample of 4-year-olds, the two preterm samples differed negligibly from each other on the measures of receptive and expressive language (<1 point). 

Also, in their *Bayley-III* study of 155 premature 2-year-olds, Toome et al. (2012) investigated the *Bayley-III* language scores. The full-term control outscored the total premature sample by 7.1 standard-score points higher on the Language Composite; the difference was 6 points on both the expressive language subtests. However, the differences were small when comparing the “26–31” group with the “<25” group: 2.9 points on language composite, 2 points on expressive language, and 3 points on receptive language. 

The small differences between preterm samples in the Toome et al. (2012) study on language ability contrast with the substantial differences observed by these same authors on both *Bayley-III* cognitive and motor scores. But, again, the results might be at least partially due to the small sample of the <25 weeks group. Ahn and Kim (2017) replicated this small language difference between samples of infants tested on the *Bayley-III* in Korea. The extremely premature sample did not differ significantly from the larger, heterogeneous sample on any *Bayley-III* score in any domain, so their results cannot address this issue. 

## 5. Purpose of the Present Study

There have been an enormous number of investigations of premature infants, especially in comparison to full-term infants, on measures of cognitive and language ability. There have been several meta-analyses. However, despite the accumulation of literally thousands of data points, notable gaps remain in the literature. There are clear implications that the degree of prematurity is directly related to cognitive loss, but those implications are mostly derived from the meta-analyses, where, for example, premature group membership is defined by the mean gestational age of each sample, and extremely and moderately premature children are combined into one group.

Moreover, most studies on prematurity focus on comparing babies born preterm versus full-term. Over the past half century, few investigators have considered the degree of prematurity as an independent variable in their studies. Only a handful of studies compared two or more samples that differed in the degree of prematurity, and those studies were hampered by methodological issues (e.g., small sample sizes, failure to control confounding variables) that compromised the interpretation of the results. Additionally, previous studies on young children have not investigated the potentially confounding effects of the age at which the children were administered the cognitive or language or motor test. For example, premature children tested as infants may differ from those tested as toddlers in view of the rapid rate of growth that occurs during the first few years of life, and, therefore, there may be differences in what constitutes intelligence in infants versus toddlers. Lastly, there have been no previous studies on the newest Bayley, namely, the fourth edition.

The purpose of this study is to begin to fill in these gaps in the literature. The *Bayley-4* manual provides data on representative samples of extremely premature, moderately premature, and full-term children. The premature and full-term samples were carefully matched on key background variables including children’s age, sex, race, and ethnicity and their parents’ education level. This matching enhanced our comparisons of the premature and full-term children, because the demographics of the two groups were very similar. Pearson Assessments provided us access to the data that would help answer the following research questions:

1. Are there mean differences in the five subtest scores between the extremely premature and moderately premature groups, after controlling for the children’s age?
2. Are there mean differences in the five subtest scores between the extremely premature and full-term control group and between the moderately premature and full-term control group, after controlling for children’s age?

To compare our findings with previous studies, we combined the two premature groups into a single heterogeneous preterm sample to address:

3. Are there mean differences in the five subtests between premature and full-term children?
4. Are there mean differences in the five subtest scores for children tested as infants (2–17 months) versus those tested as infants and toddlers (18–42 months)?
5. Does the children’s age moderate the influence of their prematurity status on their subtest scores?

## 6. Method

### Participants

Participants included 66 extremely premature children (born < 32 weeks), ages 5 to 42 months (*M* = 20.6, *SD* = 11.8); 70 moderately premature children (born 32–36 weeks, ages 2 to 42 months (*M* = 19.6, *SD* = 12.1); and 133 full-term matched control children (born 37 weeks or later) ages 2 to 42 months (*M* = 20.2, *SD* = 12.0) from the *Bayley-4* special group studies. Data were collected by Pearson Assessments to establish evidence of validity of the *Bayley-4* for specific subgroup populations (Bayley and Aylward 2019). Premature children were matched to full-term control children on several demographic variables: children’s age, sex, and race and ethnicity and their parents’ education level. 

In the premature samples, exclusionary criteria were premature children with Down syndrome, Autism Spectrum Disorder, or prenatal drug/alcohol exposure, periventricular leukomalacia, grade 4 intraventricular hemorrhage, stage III necrotizing enterocolitis, hypoxic ischemic encephalopathy with seizures, uncontrolled seizures affecting assessment engagement, oxygen dependence at time of testing, and those born small for gestational age (less than the third percentile of weight for age; Bayley and Aylward 2019). This study was approved by the Institutional Review Board under protocol number X23-0195.

Children in the *Bayley-4* special group studies were categorized into groups based on their primary classification. Twenty-one extremely premature children (32%) and six moderately premature children (9%) had a secondary classification indicated on their consent form. In the premature sample, secondary classifications included developmental delays, language delays, specific language impairments, motor impairments, hearing impairments, visual impairments, and other diagnoses. Primary and secondary classifications were not independently validated by Pearson Assessments. The first group contains both VPT (<32 weeks) and EPT (<28 weeks), while the second consists of MPT (32–33 weeks) and LPT (34–36 weeks).

Pearson Assessments matched individual full-term children from the normative sample to the children in the extremely premature and moderately premature groups. Children in the normative sample were born at full term (≥37 weeks), did not receive early childhood intervention services, and did not have developmental disorder or delay, chromosomal abnormalities affecting cognitive, language, or motor performance, infections affecting the central nervous systems, disorders secondary to prenatal exposure to toxins, central nervous system disorders, genetic or congenital disorders, errors of metabolism that affect the central nervous system, grade 3 or 4 intraventricular hemorrhage, any severe motor, cognitive, or behavioral impairments, elevated lead exposure, moderate to severe traumatic brain injury, and uncontrolled seizures affecting assessment engagement. We examined the control sample for duplicates and identified three control children who were matched to both an extremely premature and moderately premature child. We deleted these three duplicate cases from the dataset and then combined the matched controls into one control group (*n* = 133). Table 2 presents background information for the three samples.

## 7. Instrument

The *Bayley Scales of Infant and Toddler Development, Fourth Edition (Bayley-4)* is a standardized, individually administered assessment assessing the development of infants to toddlers aged 16 days to 42 months. The *Bayley-4* assesses infant development across five domains: (1) cognitive, (2) language, (3) motor, (4) social–emotional, and (5) adaptive behavior. This assessment is multifaceted through the integration of various observers, clinician observation, structured task performance, and caregiver interviews. The scoring occurs on a two-to-zero-point scale, with two points indicating mastery, one point indicating emerging skill, and zero points indicating if a skill or ability is absent (Bayley and Aylward 2019). 

The *Bayley-4* improved upon previous versions in various ways, which included updating the normative data. The caregiver participation in the assessment process is an essential component of the *Bayley-4*. According to the administration manual, the caregiver may be better at eliciting responses from the child compared with an unfamiliar examiner. Additionally, caregiver questions are included with some items so that caregivers can provide extra input in terms of the child’s response. The administration time has been reduced, and Q-Global administration and scoring is available for the *Bayley-4*. The scoring procedures have moved away from a dichotomous system to one that acknowledges that a child’s development is a fluid process. The items on the Cognitive, Language, and Motor Scales can now be scored as Mastery, Emerging, and Not Present. Furthermore, revisions made to the Bayley test aimed to improve the clinical utility of the test by including several special populations during the standardization process, such as the sample used in the present study. Revisions also included an improvement in the content across the developmental skills and added an assessment of the precursors to executive functioning (Bayley and Aylward 2019). 

For this study, the social–emotional and adaptive behavior scales were not analyzed because they were not administered to the entire population, only to participants with developmental delay classification (DD). This study considers the following subtests: cognitive, expressive language, receptive language, gross motor, and fine motor. For a more robust and fine-tuned understanding of infants’ and toddlers’ data and performance profiles, subtest scores rather than composite scores were analyzed. The subtest scores ranged from 1 to 19, with a mean of 10 and a standard deviation of 3. 

The cognitive subtest assesses one’s ability to manipulate objects, establish and hold memories, and consider early indicators of higher-order thinking. Tasks may include name recalling and identifying objects, as well as matching items by their size. The receptive communication subtest assesses both nonverbal and verbal communication styles, considering one’s ability to follow simple directions and comprehend concepts such as pronouns, plurals, and more and less. The expressive communication subtest assesses vocabulary, syntactic, and morphological skill development, consisting of tasks such as naming based on pictures, providing yes or no responses, and speaking in a sentence. The fine motor subtest assesses the integration of perceptual and motor skills, motor planning and speed, and grasping and moving objects. The gross motor subtest measures larger muscle movement, such as coordination and balance and the ability to kick a ball or walk down steps (Bayley and Aylward 2019). 

Knowing that raw data were unavailable, estimates of reliability evidence were gathered from the manual of the *Bayley-4*. Within the four special group samples, the median split-half internal consistency coefficient was 0.95 (range = 0.88–0.99; Bayley and Aylward 2019). Evidence of test–retest reliability (Corrected *r* cognitive = 0.83, Language = 0.80, Motor = 0.71) was considered via the sample of 152 typically developing children, ages 0 to 42 months, with infants and toddlers “at risk,” excluded from the analysis. Evidence of validity was also reported in the manual, showing moderate to moderately high correlations between Bayley scores and pertinent measures of cognitive and motor ability. 

### Data Analysis

Age-standardized subtest scores were analyzed via IBM SPSS version 27. Subtest scores were transformed to the standard IQ scale of *M* = 100 and *SD* = 15 to aid their interpretation. Two multivariate analyses of covariances (MANCOVA) tested for group mean differences on the five *Bayley-4* subtests. The independent grouping variable had three levels: extremely premature, moderately premature, or full-term matched control and age served as a covariate. Extremely premature children served as the reference group in one MANCOVA, and full-term matched control children were the reference group in the other MANCOVA.

To compare our findings with other studies, which mostly combined premature children across levels of prematurity severity, we combined the extremely and moderately premature groups in a two-way MANOVA. The two independent variables were prematurity status, which had two levels—premature children (*n* = 136) and full-term children (*n* = 133)—and age group, which had two levels—2 to 17 months (*n* = 128) or 18 to 42 months (*n* = 141). These age ranges were selected because they resulted in relatively similar group sizes. The interaction between prematurity status and age group was also examined for statistical significance. *T*-tests were used to probe the statistically significant interaction results.

## 8. Results

There were no missing data in the analyses. Descriptive statistics for our three samples are reported in Table 2. The subtest scores were approximately normally distributed in the three groups, and the absolute skew was below 1 and absolute kurtosis was below 1.5 (West et al. 1995). Multicollinearity was not a concern (variance inflation factor < 3.5). Box’s M test was not statistically significant in the MANCOVA model (*p* = .12) and supported the assumption of equality of covariance matrices. Box’s M test was statistically significant, however, in the MANOVA model (*p* = .01). Levene’s Test of Equality of Error Variances was examined for each dependent variable. Levene’s test was not statistically significant for the cognitive (*p* = .05), expressive communication (*p* = .17), and fine motor subtests (*p* = .06), but was statistically significant for the receptive communication (*p* = .003) and gross motor subtests (*p* = .01). Methodologists suggest MANOVA is robust to this violation of the assumption of equal covariance matrices for the two subtests, because the sample sizes of the combined premature and full-term groups were similar (*n* = 136 and *n* = 133, respectively). The more conservative Pillai’s Trace criterion was used in the MANOVA (Field 2017; Tabachnick and Fidell 2007). 

Correlations between the five subtests were statistically significant and moderate- to large-sized (*r* ranged from 0.48 between receptive communication and gross motor and receptive communication and gross motor subtests to 0.74 between receptive communication and cognitive subtests). The effect sizes were interpreted according to Cohen’s (1988) ranges. The ranges for Cohen’s *d* were small (0.2–0.49), medium (0.5–0.79), and large (≥0.80), and the ranges for partial eta squared were small (0.01–0.05), medium (0.06–0.13), and large (≥0.14).

In the MANCOVAs, the omnibus multivariate tests were statistically significant for prematurity status group (Wilks’λ = 0.73, *F* (10, 522) = 10.00, *p* < .001, ηp^2^ = 0.15, large effect size) and the continuous covariate variable age (Wilks’λ = 0.94, *F*(5, 261) = 3.32, *p* < .01, ηp^2^ = 0.06, medium effect size). There were statistically significant differences across the three groups on all five subtests, and the effect sizes ranged from medium (expressive communication ηp^2^ = 0.08, receptive communication ηp^2^ = 0.12) to large sized (cognitive ηp^2^ = 0.17, gross motor ηp^2^ = 0.20, fine motor ηp^2^ = 0.22). There was a statistically significant difference of age on the cognitive subtest only (cognitive ηp^2^ = 0.05, small effect size). 

A Bonferroni adjustment was used for all pairwise comparisons. The extremely premature children scored significantly lower than the moderately premature children on four subtests, and effect sizes were small for the expressive communication (ηp^2^ = 0.03), cognitive (ηp^2^ = 0.04), and fine motor (ηp^2^ = 0.05) subtests and medium-sized for the gross motor subtest (ηp^2^ = 0.06). There was no statistically significant difference (*p* < .05) between the extremely and moderately premature children on the receptive communication subtest. The extremely premature children scored significantly lower than the full-term children on all five subtests, and effect sizes were medium for the expressive communication (ηp^2^ = 0.08) and receptive communication (ηp^2^ = 0.11) subtests and were large for the cognitive (ηp^2^ = 0.17), gross motor (ηp^2^ = 0.20), and fine motor subtests (ηp^2^ = 0.22). In the model with the moderately premature children as the reference group, the moderately premature children scored statistically significantly lower than the full-term controls on four subtests: receptive communication (ηp^2^ = 0.04), cognitive (ηp^2^ = 0.04), and gross motor (ηp^2^ = 0.04) subtests, which all had small effect sizes, and fine motor subtest, which had a moderate effect size (ηp^2^ = 0.07). There was no statistically significant difference (*p* < .05) between the moderately premature and full-term children on the expressive communication subtest. See Table 3 for mean scores across the prematurity status groups and Table 4 for the mean differences and 95% confidence interval differences for group comparisons.

**Figure 1:**
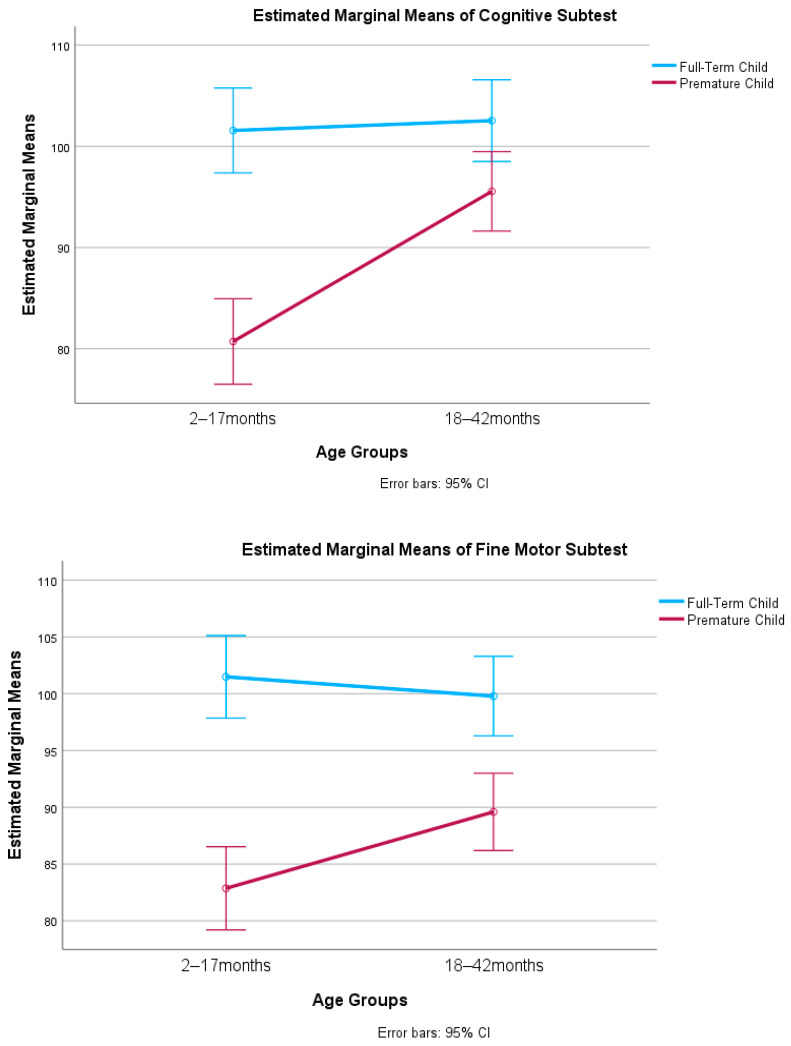
Statistically significant interactions between prematurity status and age groups.

In the MANOVA, the multivariate tests were statistically significant for prematurity status group (Pillai’s Trace = 0.23, *F*(5, 261) = 15.15, *p* < .001, ηp^2^ = 0.23 large effect), age group (Pillai’s Trace = 0.06, *F*(5, 261) = 3.54, *p* < .01, ηp^2^ = 0.06 medium effect), and the interaction between prematurity status and age (Pillai’s Trace = 0.08, *F*(5, 261) = 4.79, *p* < .001, ηp^2^ = 0.08 medium effect). There were statistically significant differences across the prematurity status groups on all five subtests, and the effect sizes were medium (expressive communication ηp^2^ = 0.06, receptive communication ηp^2^ = 0.10) and large (gross motor ηp^2^ = 0.15, cognitive ηp^2^ = 0.15, fine motor ηp^2^ = 0.19). See Table 3 for mean scores across prematurity status groups. See Table 4 for the magnitude of the differences between the various subgroups in the two multivariate analyses. There were statistically significant differences across the two age groups on two subtests, and the effect sizes were small (receptive communication ηp^2^ = 0.03, cognitive ηp^2^ = 0.05). There were statistically significant small effects of the interaction on the fine motor (ηp^2^ = 0.02) and cognitive subtests (ηp^2^ = 0.04). As shown in Figure 1, age did not appear to influence the full-term children’s fine motor and cognitive subtest scores; full-term children in both age groups performed similarly on these two subtests. However, the age of premature children influenced their scores on the fine motor and cognitive subtests. Younger premature children, aged 2 to 17 months, appeared to score lower on the two subtests than the older premature children, aged 18 to 42 months.

To further explore the premature children’s mean score differences across the two age groups, *t*-tests were used. The assumption of homogeneity of variance was not supported for the receptive communication subtest comparison (*p* < .01); thus, an adjustment to the degrees of freedom was made with the Welch–Satterthwaite method. Premature children aged 18 to 42 months scored statistically significantly higher than premature children aged 2 to 17 months on the fine motor (Cohen’s *d* = 0.42, small effect, *t*(134) = 2.46, *p* < .05), receptive communication (Cohen’s *d* = 0.49, medium effect, *t*(130.37) = 2.89, *p* < .01), and cognitive subtests (Cohen’s *d* = 0.79, large effect, *t*(134) = 4.60, *p* < .001). There was no statistically significant difference (*p* < .05) between premature children aged 2 to 17 months and 18 to 42 months on the expressive communication and gross motor subtests.

## 9. Discussion

Prematurity has undoubtedly and historically been considered an essential factor when examining an infant or child’s development, which is further echoed by the findings of this study. In short, extremely premature children demonstrated lower performances across all areas of assessed functioning compared with full-term children; moderately premature children tended to outperform extremely premature children; and premature children tested as toddlers tended to earn higher *Bayley-4* scores than premature children tested as infants. With only a few exceptions, noted in the next section, the answer to all five of our research questions is “yes.” Most importantly, these findings suggest that the degree of prematurity is critical to consider in child development and can have implications for early intervention and the consideration of specific patterns of abilities, as discussed in later sections.

### 9.1. Major Findings

Table 3 provides a snapshot of the most important findings of our investigation:

(a) The *Bayley-4* cognitive and motor subtests were the most potent discriminators between full-term and preterm samples. The full-term sample scored substantially higher than the extremely preterm sample on the cognitive, gross motor, and fine motor subtests when compared with the extremely premature children (19 points), moderately premature (9 points), and combined premature samples (14 points).
(b) By contrast, prematurity had a notably weaker effect on the development of language abilities, both expressive and receptive. Full-term children significantly outscored preterm children on the language subtests, but the differences were smaller when compared with the extremely premature (13–14 points), moderately premature (7 points), and combined premature samples (10 points).
(c) The moderately premature sample scored significantly higher than the extremely premature sample on all five subtests, but there were differences by domain. Once more, cognitive and motor abilities were the most affected by degree of prematurity (a 9–10-point advantage for the moderately preterm group) versus a 6–7-point difference for the two language subtests.
(d) The age at which children are assessed on the *Bayley-4* affected the degree to which they displayed developmental deficits. When tested as infants (2–17 months), premature children showed a striking intellectual deficit on the *Bayley-4*. They averaged 80 on the cognitive subtest (Table 2). By comparison, the premature children who were tested as toddlers (18–42 months) scored 14 points higher. Though these findings are based on separate samples of infants and toddlers, the implication (requiring verification) is that there is a good amount of cognitive growth that occurs as premature infants enter toddlerhood. By contrast, the fine motor and receptive communication subtests produced only moderately higher scores for preterm children tested as toddlers versus infants (7 pts.), and the gross motor and expressive communication subtests did not yield significant differences between the two age groups.

### 9.2. Cognitive and Language Domains

Interestingly, in the cognitive domain, prior research has rarely considered the role of the degree of prematurity when examining intellectual abilities, instead focusing more on comparing full-term with preterm peers or recruiting subjects within a specific time frame of degree of prematurity. Despite this virtual lack of prior data, the present findings do, in general, align with the widely held belief that less gestational time leads to more concerns with intellectual functioning (Allotey et al. 2017), with present findings indicating differences in the cognitive abilities of extremely versus full-term (large effect size), moderately versus full-term (small effect size), and extremely versus moderately premature (small effect size) children. Synthesizing the current findings with prior research, the impact of the degree of prematurity on cognitive ability supports prior findings that the gestational age at birth contributes to between 38–48% of the variance in IQ (Allotey et al. 2017). Thus, cognitive abilities may be negatively affected by a baby’s decreased time in the womb, with additional time allowing for further growth of critical areas of the brain. In sum, the present cognitive findings contribute to this growing area of research, postulating that degree of prematurity is critical to consider when assessing cognitive skills with the *Bayley-4*. In addition, understanding the degree of prematurity of a baby may lend additional information on potential cognitive skill deficits and related supports that schools or birth-to-three programs may consider implementing.

Similar to previous research, our findings indicated that the language scales yielded the least amount of difference in developmental scores for full-term versus preterm infants. Across the subtests of the *Bayley-III*, Månsson and Stjernqvist (2014) found that extremely premature children had a minor score difference compared with full-term peers (at 2.5 years old), although the scores were still significantly different. Zimmerman (2018) indicated that very preterm children scored 0.78 to 0.99 standard deviations below their preterm peers in total language when tested at preschool and kindergarten ages. These children looked more “average” than expected in these primary years. This research has substantial implications for educational placement, considering that the educational criteria for developmental delays typically depend on one to two standard deviation delays for defining the degree of language delay in the school and clinical settings. 

### 9.3. Gross and Fine Motor 

In examining fine motor skill development, previous research has revealed significant differences between full-term and preterm births (de Kieviet et al. 2009; Ribeiro et al. 2017), potentially having a lasting impact on fine motor skill development into adolescence (Thomas et al. 2017). Similarly, gross motor outcomes for premature infants are often delayed, frequently until early grade school (Ribeiro et al. 2017) and into later development (de Kieviet et al. 2009). While prior research (e.g., van Haastert et al. 2006) has considered the differences in gross and fine motor skills between full and preterm infants, this study contributes additional information regarding the severity of the impact based on the child’s degree of prematurity. 

## 10. Age Interactions and Neurological Considerations

Next, the interactions between age and domain area were explored. Significant interactions were noted for fine motor and cognition. Additionally, to examine the impact of age on the development of babies born prematurely, the present findings suggested that toddlers (18 to 42 months) outperformed infants (2 to 17 months) across the fine motor (small effect) and cognition (large effect) domains. The findings suggest that with time, the development in these arenas of functioning appears to recuperate, which concurs with prior research around central nervous system maturation and motor skill development (Bartlett 2000; Bartlett and Lucy 2004), as well as cognitive theories of neuroplasticity’s impact on early development (Luciana 2003). These findings also conform with research suggesting that the reduced skills of children born prematurely may recover to a certain extent (Bartlett 2000; Bartlett and Fanning 2003; Grigoroiu-Serbanescu 1981). This finding also adds to Grigoroiu-Serbanescu’s (1981) postulation of a “recovery period”, where individuals born premature can make significant gains in cognitive abilities. This growth period may also indicate more plasticity for cognitive development early in the infant’s development, providing divergent evidence from Breeman et al. (2015), who suggested that IQ may be more stable for those born prematurely.

## 11. Contribution of New Knowledge

The current study adds new findings to the body of research examining the impacts of prematurity on cognitive, language, and motor skill development. Specifically, we compare two age groups at three levels of gestation. Previous studies have invariably focused on a single preterm sample, tested at a single age (e.g., Anderson 2010; Caravale 2005; Censullo 1994; Gray et al. 2006) or occasionally used a low birthweight as a proxy for prematurity (e.g., Munck et al. 2010). The present study provides data on the age interaction, demonstrating a possible growth in cognitive, language, and motor functioning and skill development between infancy and toddlerhood, regardless of the degree of prematurity. Second, previous research has focused more on the development of cognitive abilities; the current study adds essential considerations for language development and fine and gross motor skills. These implications are influential, since recent research has found these skill areas to be interrelated. As an illustration, Kobaş et al. (2022) found that motor development, visual processing, and language skills were significantly related to one another for preterm infants.

Limited prior research has examined these skill areas across varying degrees of prematurity. The studies that included the degree of prematurity as a variable had notable methodological issues (Ahn and Kim 2017; Toome et al. 2012; Woodward et al. 2009). And, of great importance was the careful matching of our premature and full-term samples on important background variables (i.e., children’s age, sex, and race and ethnicity, and their parents’ education level). Given this careful attention towards matching, the demographic characteristics of the premature and full-term groups are very similar.

Additionally, this is the first study to date that utilizes the most recent version of the *Bayley Scales of Infant and Toddler Development*, the *Bayley-4*, with a premature population. Notably, the Standards for Educational and Psychological Testing indicate that “It is commonly observed that the validation process never ends…” (American Educational Research Association et al. 2014; pp. 21–22), and our study utilizes the updated normative data on premature infants and toddlers. The revisions of the *Bayley-4* aimed to improve the utility of the test with clinical populations, including infants and toddlers born prematurely. It is important to get this knowledge into the literature base outside of test manuals. Furthermore, given the other updates to the test (i.e., improved item content, reduced administration times, greater caregiver involvement, etc.), our study demonstrates that the *Bayley-4* is a useful tool when assessing premature infants and toddlers and should be utilized in place of its predecessors.

## 12. Impact beyond Preschool

An accurate assessment of language and cognitive skills during infancy and toddlerhood is essential to developing early intervention plans and can provide insights into the long-term outcomes for these children. Research on the long-term outcomes for individuals born prematurely indicates impairments across cognitive, academic, and behavioral domains that persist beyond toddlerhood (e.g., Allotey et al. 2017). It has been shown that, for individuals born preterm (<37 weeks GE), deficits in memory, attention, processing speed, and visuospatial abilities—which are evident in infancy—persist into early adolescence (Rose et al. 2011). Recent research has corroborated these findings. At ages 8 to 15 years, significant deficits in visuospatial abilities were evident among children born prior to 37 weeks GE (Butti et al. 2020). In longitudinal research, children who were born very prematurely (GA < 30 weeks) were found to exhibit attentional difficulties, including irregular attention patterns at ages 7 and 13 (Bogičević et al. 2021). Based on a meta-analysis of 18 studies that used behavioral outcomes, Allotey et al. (2017) found that babies born prematurely displayed significantly more behavioral problems than those born full-term at primary and secondary school ages and were much more likely to be diagnosed with attention-deficit/hyperactivity disorder (ADHD). At ages 5.5 and 18, children born <36 weeks GE performed significantly lower than their full-term peers on working memory and cognitive flexibility tasks (Stålnacke et al. 2019). 

A meta-analysis conducted between 2003 and 2016 with children aged 5 to 18 years concluded that prematurely born (GA < 37 weeks) children earned significantly lower standardized academic achievement scores in the areas of arithmetic, reading, and spelling (0.77, 0.44, and 0.52 SDs lower, respectively) and were three times more likely to receive special education than full-term controls (Twilhaar et al. 2018). Similar deficits in math, reading, and spelling were observed in primary school by Allotey et al. (2017) in their meta-analysis.

Further, at 24 months of age, test scores of children born moderately preterm predicted Full-Scale IQ, Verbal IQ, and behavior at six years of age (Bogičević et al. 2019). The Luttikhuizen dos Santos et al. (2013) study, cited earlier, showed emphatically that cognitive and language development on the Bayley test (summarized as the MDI) was an excellent predictor of future IQ (0.61 correlation across 14 studies). 

But even the notable relationships between Bayley scores and long-term outcomes account for less than 40% of the variance, reminding us that an array of variables affect test performance apart from premature status. For example, Adams-Chapman et al. (2013), in their study on preterm infants, found that other factors, such as feeding problems, were associated with lower language and cognitive outcomes on the Bayley test. Also, there is substantial research suggesting that the practice of administering corticosteroids to facilitate respiration in preterm children is associated with later cognitive delays (Räikkönen et al. 2020). And recent research suggests that a certain brand of incubator used with preterm children may impact hearing (Bertsch et al. 2020). 

## 13. Theoretical Underpinnings

Rooted deeply in Arnold Sameroff’s Transactional Model of Development, the “plastic character” of the individual is constantly influenced by their environments while, at the same time, they seek to organize and structure the world they inhabit. Knowing this, the time-honored nature/nurture debate suggests that a baby born preterm, who endured no other traumas and was raised in a suitable home environment, may have typical outcomes compared with peers born full-term as babies (Sameroff 1975). In Transactional Theory, development is not determined simply by one traumatic event, but instead is an accumulation of development across time within the context of environmental interaction (Sameroff 1975). Knowing this, Sameroff suggests that when seeking to understand developmental processes, researchers and clinicians should continually assess the transactions between the environment and the child instead of a continuous assessment of the child in singularity. Sameroff (1975) provides examples in his numerous texts on the theory, suggesting that obstetricians should work to support the expecting individual (and, we argue, the individual’s partner as well) to identify and recommend support for any environmental or personal factors that may later impact the baby, such as anxiety. 

The intersections of biology and environment are also critical in other modern-day theories, such as The Adverse Childhood Experiences (ACE) perspective (Felitti et al. 1998; McLaughlin 2017). Extending these theories and works to the topic of prematurity, such theoretical perspectives argue that beyond what an individual may be biologically predisposed to, children may grow into fully functioning adults because of, and despite, their premature birth status. This perspective is vital in offering a more positive and hopeful outlook for young infants, children, and adults impacted by premature birth. 

## 14. Limitations and Directions for Future Research

The present study should be considered within the context of its limitations. For one, children categorized as extremely or moderately premature were carefully matched to each other, and to the control samples, on important background variables. But Pearson Assessments cannot be certain exactly which children had secondary diagnoses such as ADHD or language delay. Within the three subsamples, there are likely unknown secondary classifications or diagnoses present in the one full-term and two preterm samples. Some standardization examiners report co-morbidities, others do not; this variability from examiner to examiner is a common criticism of normative and validation samples of all clinical tests. It is plausible that co-morbidities are more prevalent in the preterm samples, especially the “Extremely” group, in keeping with the findings of this study that the degree of prematurity relates to the degree of impairment across domains. Similarly, secondary classifications are more likely among the toddlers than the infants in this study, because 2- and 3-year-olds are far more likely to be referred for evaluation than infants. In fact, secondary classifications are important to consider given the potential for confounding variables contaminating research data; simultaneously, this general topic speaks to the challenges that school psychologists routinely face when differentiating between primary and secondary educational classifications to provide accurate services. 

A second limitation concerns the important new scales that are included in the *Bayley-4*, namely the social–emotional and adaptive behavior scales. Data from the new scales were not available for any of the validity samples, including the preterm groups. Investigating the cognitive, language, and motor skill domains helped answer important questions about the effects of prematurity, but how wonderful it would have been if we had been able to investigate the adaptive behavior and socio–emotional domains as well. These data would have been helpful given the long-term concerns that children and adolescents born preterm may have in their social, emotional, and adaptive functioning. In addition, these data would likely have been helpful for future educational and transition planning when considering support for children with an IEP. 

Finally, it is crucial to note that the data analyzed were cross-sectional. When considering future directions for research, a longitudinal design that follows preterm infants and toddlers through childhood and beyond would be ideal. Initial assessments would be with all scales of the *Bayley-4*; subsequent assessments would be with state-of-the-art measures of intelligence, language ability, motor coordination, adaptive behavior, and socio-emotional functioning. The degree of prematurity would be a key independent variable. With this design, a more nuanced picture of what time and intervention can provide to a child’s growth could be attested to more directly. Furthermore, age and the degree of prematurity may help establish predictions in services and progress monitoring. 

## 15. Conclusions

Consistent with the large body of literature on the cognitive, language, and motor deficits of children born preterm, the present findings—based on carefully matched samples of infants and toddlers—reveal deficits across domains. This study’s design, however, adds to the literature by providing convincing data that the degree of prematurity matters, and that it matters a great deal. Children born moderately prematurely outperformed those born extremely prematurely on every subtest in every domain. The cognitive and motor subtests produced the largest differences. As has been found in some previous studies, language development, both expressive and receptive, appears to be far less vulnerable to the impact of prematurity than cognitive and motor abilities. This study also demonstrated that the age at which a child is tested is integrally related to their *Bayley-4* test performance. Children assessed as infants showed greater deficits than those tested as toddlers in two domains (cognitive and fine motor subtests), with cognitive ability producing the most sizable discrepancy. Though these data were cross-sectional, these results are strongly indicative that growth occurs across domains as premature infants get older, a finding that is consistent with previous research, neurological development, and transactional theory. Further, these findings add to a growing body of international research examining the impact of premature birth, concurring with many of the prior findings around the influence on cognitive (e.g., Bogičević et al. 2019; Breeman et al. 2015; Cheong et al. 2017; Kaul et al. 2021; Mangin et al. 2017; Maggi et al. 2014; Serenius et al. 2016; Potharst et al. 2013), motor (e.g., De Rose et al. 2013; Lee et al. 2004; Lorefice et al. 2015; Maggi et al. 2014; Potharst et al. 2013; Van Hus et al. 2014), and language development (e.g., Da Costa Ribeiro et al. 2016; Nguyen et al. 2018; Putnick et al. 2017; Reidy et al. 2013; Tulviste et al. 2020; Varela-Moraga et al. 2023; Verreschi et al. 2020). 

Overwhelmingly, these data support the argument that time in the womb is vital for long-term development. However intriguing, this finding does not lead to many practical outcomes for practitioners working with children and families after the children’s birth. When shifting to a more practical lens, this study’s findings are far more consistent with early intervention than with the once widely used wait-to-fail model (Bayley and Aylward 2019). Given the historical precedent and the present state of the research supporting the effectiveness and efficiency of very early intervention (Campbell et al. 2001; Craig and Campbell 1984)—coupled with the U.S. federal policy mandating the implementation of early intervention (birth-to-three, Individuals with Disabilities Education Improvement Act 2004)—these data support the urgency for intervention in infancy, across multiple domains of functioning, given the widespread deficits that premature babies are likely to face. As noted previously, those deficits extend to noncognitive domains. Children born prematurely are significantly more likely to be diagnosed with ADHD as children than those born full-term; indeed, the odds increase threefold for samples of children who were born moderately, or very, prematurely (Allotey et al. 2017).

Additionally, these data may be helpful for practitioners and assessors of infants and their families in order to consider the degree of prematurity during the initial interview and at the data collection stage. Paying attention to the number of weeks a child was born early may affect decision making about interventions. This perspective may help in designing a comprehensive treatment plan for families receiving birth-to-three services, as well as inform the comprehensive assessment of children to prepare for widespread deficits across domains of functioning. 

From a hopeful perspective, the present study emphasizes the recovery in skill abilities across development. When children are tested at different ages, some of the deficits observed during infancy may have been recovered. Early intervention and working with children during critical periods of development may help recoup some of the lost skills. These findings are a testament to the intervention of professionals in the birth-to-three arenas.

## Figures and Tables

**Table 1:** Cognitive scores earned by preterm versus full-term children tested at ages 1.5 to 4 years of age.

Author	Effect Size (95% CI)	IQ Mean Difference	Total N	Premature N	Full-Term N	Classification	Age at Testing	Scale
Anderson (2010)	0.85 (0.65, 1.05)	12.75	413	211	202	Large	2 years, 0 months corrected age	*Bayley-III*
Baron et al. (2012)	Very Preterm 0.35 (0.12, 0.58) Extremely Preterm 0.88, (0.54, 1.21)	5.25 Very Preterm 13.20 Extremely Preterm	396	196 Very Preterm 52 Extremely Preterm	121	Small Large	3 years, 0 months, 3 years, 11 months	DAS-II CGA
Bode et al. (2009)	Cohort 1 0.98 (0.69, 1.26) Cohort 2 0.42 (0.20, 0.63)	14.70 Cohort 1 6.30 Cohort 2	542	106 Cohort 1 167 Cohort 2	269	Large Small	2 years, 0 months corrected age	*Bayley-I*
Breeman et al. (2015)	0.97 (0.78, 1.16)	14.55	458	230	228	Large	4 years, 0 months	*Columbia Mental Maturity Scale*
Caravale (2005)	0.96 (0.42, 1.49)	14.40	60	30	30	Large	3 years, 0 months–4 years, 0 months	*Stanford-Binet*
Censullo (1994)	0.87 (0.43, 1.31)	13.05	87	40	47	Large	2 years, 0 months	*Bayley-I*
Gray et al. (2006)	0.61 (0.33, 0.89)	9.15	204	100	104	Medium	2 years (+/−2 weeks) corrected age	*Bayley-II*
Munck et al. (2010)	0.59 (0.39, 0.80)	8.85	374	182	192	Large	2 years of corrected age (CA) (from) 1 week to +1 month)	*Bayley-I*
Sajaniemi et al. (1998)	1.31 (0.97, 1.65)	19.65	160	80	80	Large	1 year, 8 months–2 years, 4 months corrected age	*Bayley-I*
Toome et al. (2012)	Very Preterm 0.56 (0.32, 0.79) Extremely Preterm 1.11 (0.59, 1.62)	8.40 Very Preterm 16.65 Extremely Preterm	308	17 Extremely Preterm 138 Very Preterm	153	Medium Large	2 years of corrected age 2 years (+/−1 month)	*Bayley-III*
Woodward et al. (2009)	Very Preterm 0.67 (0.34, 0.98) Extremely Preterm 0.74 (0.37, 1.10)	10.05 Very Preterm 11.10 Extremely Preterm	212	62 Very Preterm 43 Extremely Preterm	107	Medium Large	4 years (+/−2 weeks) corrected age	*WPPSI-R*

Effect sizes for studies assessing IQ below age four. Note: Cohen’s (1988) classification of effect sizes is consulted for small (0.2–0.49), medium (0.5–0.79), and large (≥0.80) effect sizes.

**Table 2:** Demographics for three groups and total sample.

	Full-Term Control (≥37 Weeks) (*n* = 133)	Extremely Premature (<32 Weeks) (*n* = 66)	Moderately Premature (32–36 Weeks) (*n* = 70)	Total Sample (*n* = 269)
Gender				
Female	77 (57.9%)	37 (56.1%)	44 (62.9%)	158 (58.7%)
Male	56 (42.1%)	29 (43.9%)	26 (37.1%)	111 (41.3%)
Race/Ethnicity				
Asian	2 (1.5%)	2 (3.0%)	1 (1.4%)	5 (1.9%)
Black/African American	20 (15.0%)	19 (28.8%)	4 (5.7%)	43 (17.8%)
Hispanic	20 (15.0%)	6 (9.1%)	13 (18.6%)	39 (14.5%)
White	83 (62.4%)	35 (53.0%)	49 (70.0%)	167 (62.1%)
Other	8 (6.0%)	4 (6.1%)	3 (4.3%)	15 (5.6%)
Parent’s Level of Education				
0-12 years of school, no diploma	5 (3.8%)	4 (6.1%)	1 (1.4%)	10 (3.7%)
High school diploma or equivalent	14 (10.5%)	6 (9.1%)	8 (11.4%)	28 (10.4%)
Some college/technical school/associate degree	39 (29.3%)	16 (24.2%)	23 (32.9%)	78 (29.0%)
Bachelor’s degree	75 (56.4%)	40 (60.6%)	38 (54.3%)	153 (56.9%)
Children’s Age in Months	*M(SD)*	*M(SD)*	*M(SD)*	*M(SD)*
	20.2 (12.0)	20.6 (11.8)	19.6 (12.1)	20.1 (11.9)

Note. Percentages may not sum to 100% due to rounding.

**Table 3:** Subtest standard scores by group.

	Extremely Premature (*n* = 66) (<32 Weeks)	Moderately Premature (*n* = 70) (32–36 Weeks)	Full-Term Control (*n* = 133) (≥37 Weeks)	Combined Premature (*n* = 136) (<37 Weeks)	Combined Premature 2–17-Month-Olds (*n* = 63)	Combined Premature 18–42-Month-Olds (*n* = 73)
	*M (SD)*	*M (SD)*	*M (SD)*	*M (SD)*	*M (SD)*	*M (SD)*
Cognitive	83.6 (21.1)	93.4 (18.1)	102.1 (15.0)	88.7 (20.1)	80.7 (18.5)	95.5 (19.0)
Receptive Communication	87.7 (17.7)	94.4 (18.6)	102.2 (15.7)	91.1 (18.4)	86.4 (14.7)	95.1 (20.3)
Expressive Communication	88.4 (16.7)	96.1 (17.8)	100.7 (16.1)	92.4 (17.6)	91.1 (17.4)	93.5 (17.9)
Fine Motor	81.9 (16.8)	90.8 (14.5)	100.6 (13.4)	86.5 (16.2)	82.9 (16.6)	89.6 (15.4)
Gross Motor	81.9 (15.8)	92.4 (16.1)	100.0 (14.0)	87.3 (16.8)	85.3 (18.0)	89.0 (15.6)

Note. *Bayley-4* subtest scaled scores have a mean of 10 and a standard deviation of 3. We transformed the subtest scaled scores to standard scores (*M* = 100 and *SD* = 15) to aid their interpretation. Our statistical transformations match the conversion table in the *Bayley-4* technical manual (Bayley and Aylward 2019).

**Table 4:** Group comparison of mean differences and 95% confidence intervals for differences.

	Full-Term (*n* = 133) vs. Combined Premature (*n* = 136)	Full-Term (*n* = 133) vs. Extremely Premature (*n* = 66)	Full-Term (*n* = 133) vs. Moderately Premature (*n =* 70)	Moderately Premature (*n* = 70) vs. Extremely Premature (*n =* 66)	Combined Premature 18–42 Months (*n* = 63) vs. Combined Premature 2–17 Months (*n* = 73)
Cognitive	13.9 (9.8–18.0) *	18.6 (12.4–24.8) *	8.5 (2.4–14.5) *	10.1 (3.1–17.2) *	14.8 (8.4–21.2) *
Receptive Communication	11.4 (7.3–15.5) *	14.6 (8.5–20.8) *	7.8 (1.7–13.8) *	6.9 (−0.1–13.8)	8.7 (2.6–14.8) *
Expressive Communication	8.3 (4.2–12.4) *	12.3 (6.3–18.4) *	4.5 (−1.5–10.4)	7.8 (0.9–14.7) *	2.4 (−3.6–8.4)
Fine Motor	14.4 (10.9–18.0) *	18.8 (13.5–24.0) *	9.8 (4.6–14.9) *	9.0 (3.0–15.0) *	6.7 (1.3–12.2) *
Gross Motor	12.8 (9.1–16.6) *	18.1 (12.7–23.6) *	7.5 (2.2–12.9) *	10.6 (4.4–16.8) *	3.7 (−2.0–9.3)

Note. Asterisks indicate that mean differences are statistically significant at *p* < .05. A Bonferroni adjustment for multiple comparisons was used. *Bayley-4* subtest scaled scores have a mean of 10 and a standard deviation of 3. We transformed the subtest scaled scores to standard scores (*M* = 100 and *SD* = 15). We calculated the mean differences using the standard scores to aid their interpretation. Our statistical transformations match the conversion table in the *Bayley-4* technical manual (Bayley and Aylward 2019).

## Data Availability

Standardization data from the Bayley Scales of Infant and Toddler Development™, Fourth Edition (Bayley-4). Copyright © 2019 NCS Pearson, Inc. Data used with permission. All rights reserved. NCS Pearson, Inc., Bloomington, IN, USA.
