# Correction: Winter et al. (2023). Does the Degree of Prematurity Relate to the Bayley-4 Scores Earned by Matched Samples of Infants and Toddlers across the Cognitive, Language, and Motor Domains? *Journal of Intelligence* 11: 213

**DOI:** 10.3390/jintelligence13080091

**Published:** 2025-07-23

**Authors:** Emily L. Winter, Jacqueline M. Caemmerer, Sierra M. Trudel, Johanna deLeyer-Tiarks, Melissa A. Bray, Brittany A. Dale, Alan S. Kaufman

**Affiliations:** 1 School of Health Sciences Clinical PsyD Program, Touro University, New York, NY 10036, USA; 2 Department of Educational Psychology, University of Connecticut, Storrs, CT 06268, USA; jacqueline.caemmerer@uconn.edu (J.M.C.); melissa.bray@uconn.edu (M.A.B.); alan.kaufman@uconn.edu (A.S.K.); 3 Department of Occupational and Environmental Medicine, University of Connecticut School of Medicine, Farmington, CT 06030, USA; sierra.trudel@uconn.edu; 4 School-Clinical Child Psychology Program, Pace University, New York, NY 10038, USA; jdeleyertiarks@pace.edu; 5 Department of Special Education, Ball State University, Muncie, IN 47306, USA; badale@bsu.edu

## Text Correction

There was an error in the original publication (Winter et al. 2023) concerning the results presented in the abstract.

A correction has been made to the abstract:

Extremely premature children scored significantly lower than moderately premature children on four subtests (expressive communication, cognitive, fine motor, gross motor), and both preterm groups were significantly outscored by the full-term sample (across all subtests in extremely preterm and across four subtests in moderately preterm). In most of the group comparisons, the cognitive and motor subtests yielded larger differences than the expressive and receptive communication subtests. A follow-up MANOVA was conducted to examine full-term versus preterm discrepancies on the five subtests for infants (2–17 months) vs. toddlers (18–42 months). For that analysis, the two preterm groups were combined into a single preterm sample, and a significant interaction between the age level and group (full-term vs. preterm) was found. Premature infants scored lower than premature toddlers on the fine motor and cognitive subtests.

In addition, a change has been introduced to the Data Availability Statement; the section has been corrected to the following:

Restrictions apply to the availability of these data. Data were obtained from Pearson Assessments and are available via from https://www.pearsonassessments.com/forms/standardization-data-license-requests.html with the permission of Pearson Assessments. Standardization data from the Bayley Scales of Infant and Toddler Development™, Fourth Edition (Bayley-4). Copyright © 2019 NCS Pearson, Inc. Data used with permission. All rights reserved. NCS Pearson, Inc., Bloomington, IN, USA.

The authors state that the scientific conclusions are unaffected. This correction was approved by the Academic Editor. The original publication has also been updated.
